# Orthodontic Mini-Implants for Interim Tooth Replacement in Growing Patients with Hypodontia: A Narrative Review

**DOI:** 10.3390/jcm14144963

**Published:** 2025-07-14

**Authors:** Oskar Komisarek, Jacek Kwiatkowski, Natalia Szczypkowska, Łukasz Banasiak, Paweł Burduk

**Affiliations:** 1 Department of Otolaryngology, Phoniatrics and Audiology, Faculty of Medicine, Collegium Medicum, Nicolaus Copernicus University in Toruń, 85-067 Toruń, Poland; pburduk@cm.umk.pl; 2 Department of Otolaryngology, Laryngological Oncology and Maxillofacial Surgery, University Hospital, 85-094 Bydgoszcz, Poland; jacekkwiat@poczta.onet.pl; 3 Students Scientific Society of Maxillofacial Surgery, Poznan University of Medical Sciences, 61-701 Poznań, Poland; klotolary@cm.umk.pl; 4 The Regional Specialist Hospital in Olsztyn, 10-561 Olsztyn, Poland; drbanasiak@gmail.com

**Keywords:** orthodontic mini-implants, miniscrew, hypodontia, tooth agenesis, growing patients, interim prosthetic rehabilitation, alveolar bone preservation

## Abstract

**Background**: Tooth agenesis, particularly hypodontia, poses a clinical and esthetic challenge in growing patients due to limitations in definitive implant placement before skeletal maturity. Traditional solutions such as removable prostheses or orthodontic space closure often fail to provide adequate long-term stability, function, and tissue preservation. In recent years, orthodontic mini-implants have emerged as a promising interim solution. This narrative review aims to synthesize current clinical evidence on the use of orthodontic mini-implants as temporary prosthetic abutments in children and adolescents with hypodontia or post-traumatic tooth loss. **Methods**: A literature search was conducted using PubMed and Google Scholar databases, covering studies published between January 2004 and March 2025. Inclusion criteria were clinical reports involving skeletally immature patients with congenital or traumatic tooth loss treated with mini-implants, with mandatory radiographic diagnostics and outcome data. Data extracted included patient demographics, etiology, implant site, imaging, follow-up, complications, and outcomes. A total of 17 studies comprising 42 cases were analyzed and summarized in tabular form. **Results**: Patients aged 6 to 16 years were treated primarily for agenesis of maxillary lateral or central incisors. The mean follow-up duration was 36.9 months. CBCT was used in 28.6% of cases. Mini-implants demonstrated high clinical success with stable soft tissue contours and preservation of alveolar volume. Complications were reported in 21.4% of cases and included crown debonding, minor infraocclusion, soft tissue irritation, and rare instances of osseointegration. **Conclusions**: Orthodontic mini-implants may provide a minimally invasive and reversible approach to interim tooth replacement in growing patients. Preliminary evidence suggests favorable outcomes in terms of stability, esthetics, and tissue preservation, but further prospective research is needed to validate their long-term effectiveness and standardize clinical application.

## 1. Introduction

Hypodontia, defined as the congenital absence of one to six permanent teeth, is one of the most prevalent developmental anomalies affecting the dentition. It occurs more frequently in the permanent dentition than in the primary one, with the most commonly missing teeth being the maxillary lateral incisors, second premolars, and mandibular central incisors [1,2]. The prevalence of hypodontia varies depending on ethnicity and population, typically ranging between 1% and 10% [3]. Depending on the number of missing teeth, dental agenesis can be further classified into hypodontia (1–5 missing teeth), oligodontia (6 or more missing teeth), and anodontia (complete absence of teeth) [4].

The etiology of hypodontia is multifactorial and includes genetic, environmental, and epigenetic factors. While hypodontia may present as an isolated finding, it is frequently associated with various syndromes, such as ectodermal dysplasia, Down syndrome, and cleft lip and palate [5].

The absence of teeth, especially in the anterior region, may lead to significant clinical consequences, including impaired esthetics, phonetic difficulties, compromised masticatory function, and alveolar bone deficiency. In growing patients, congenital absence of anterior teeth has a profound psychosocial impact and may result in reduced self-esteem and social withdrawal [6].

Treatment of hypodontia in growing individuals presents a therapeutic challenge. Osseointegrated prosthetic implants are generally contraindicated until craniofacial growth has been completed to avoid complications such as infraocclusion, implant ankylosis, and interference with alveolar development [7]. Conventional interim solutions, such as removable prostheses, orthodontic space closure, or adhesive bridges, are commonly used but have several limitations including reduced esthetic outcome, poor retention, and inadequate stimulation of the alveolar ridge [8].

Orthodontic mini-implants were initially developed for anchorage purposes but are now increasingly used as temporary prosthetic abutments in growing patients. Due to their small diameter and the absence of osseointegration, they offer the advantage of being removable after growth completion without compromising future implant placement [9,10]. In selected cases, mini-implants allow for immediate functional and esthetic rehabilitation, support alveolar bone volume, and prevent soft tissue collapse [11,12]. The aim of this narrative review is to summarize the current literature regarding the use of orthodontic mini-implants as a temporary prosthetic solution in children and adolescents with hypodontia.

## 2. Materials and Methods

This narrative review was conducted to synthesize current clinical evidence on the use of orthodontic mini-implants as temporary prosthetic solutions in children and adolescents with hypodontia or traumatic tooth loss during skeletal immaturity. The narrative format was selected due to the heterogeneity of available data, predominance of descriptive case studies, and absence of randomized controlled trials, which precluded the feasibility of conducting a systematic review or meta-analysis. Nevertheless, a structured multi-reviewer screening process was applied to minimize bias during article selection.

### 2.1. Search Strategy

A comprehensive literature search was carried out using the PubMed and Google Scholar databases, covering the period from January 2004 to March 2025. The search terms included the following: “mini dental implants”, “temporary anchorage device”, “TAD”, “hypodontia”, “tooth agenesis”, “growing patient”, and “alveolar bone preservation”. Boolean operators (AND, OR) were applied to refine results. Only studies published in English and available in full-text format were considered. The final search was performed on 15 March 2025.

### 2.2. Eligibility Criteria

#### 2.2.1. Inclusion Criteria

- Clinical studies reporting the use of orthodontic mini-implants in skeletally immature patients with congenital tooth agenesis or post-traumatic tooth loss.
- Availability of the full article in English.
- Detailed description of the clinical protocol, including radiographic imaging (mandatory use of at least one of the following: panoramic radiographs, periapical radiographs, cephalometric radiographs, or CBCT).
- Documentation of follow-up and outcomes related to implant stability, esthetic function, or alveolar bone response.

#### 2.2.2. Exclusion Criteria

- Studies involving skeletally mature or adult patients.
- Articles lacking clinical data (e.g., technical notes, reviews, or theoretical analyses).
- Studies omitting radiographic assessment as part of diagnosis or follow-up.
- Reports with incomplete data on follow-up duration, outcomes, or complications.

#### 2.2.3. Study Selection and Data Extraction

Four independent reviewers screened titles and abstracts to identify eligible studies. Full texts were assessed against the inclusion and exclusion criteria. Disagreements were resolved through discussion. From each study, the following variables were extracted: number of cases, patient age and sex, etiology of tooth loss, anatomical site of implant placement, implant type and dimensions, prosthetic design, duration of follow-up, radiographic modalities used, complications, and reported clinical outcomes. To minimize selection bias, four independent reviewers (O.K., J.K., N.S., Ł.B.) screened all titles and abstracts. Full-text articles were evaluated using predefined inclusion and exclusion criteria. Any disagreements were resolved through consensus discussion. Although this is a narrative review, this multi-reviewer approach aimed to enhance transparency and ensure consistency in the study selection.

The collected data were organized descriptively. Due to variations in the study design and outcome measures, a quantitative synthesis was not performed.

## 3. Results

Seventeen eligible publications describing the use of orthodontic mini-implants for interim prosthetic rehabilitation in growing patients were included in this review. Collectively, these reports accounted for 42 individual clinical cases. Patients were aged between 6 and 16 years, with most in their early teens. Among 27 cases with reported sex, 17 were male and 10 were female.

All extracted data were organized and presented in a structured table (Table 1) to facilitate comparative analysis across cases.

Tooth loss was mainly due to congenital hypodontia (10 studies), with additional cases linked to trauma (n = 4), ectodermal dysplasia (n = 2), or mixed causes (n = 1).

The most frequently replaced units were the maxillary lateral incisors (teeth 12 and 22) and central incisors (11 and 21). Less commonly, mini-implants were used to substitute mandibular premolars, deciduous molars, or first permanent molars.

The follow-up period ranged from 12 to 99 months, with a mean observation time of 36.9 months. Across all cases, treatment outcomes were reported as favorable, with high rates of implant stability, prosthetic retention, and functional and esthetic satisfaction. Several studies documented vertical bone maintenance or gain, indicating a possible role for mini-implants in preserving alveolar ridge volume during craniofacial development.

Imaging protocols varied, with panoramic/periapical radiographs used most often. CBCT was performed in 28.6% of cases, but no standard imaging approach was followed. Adverse events were reported in 9 out of the 42 cases (21.4%), with complications distributed across seven studies. These included the following:

- Repeated debonding of temporary crowns and traumatic explantation with associated alveolar damage (n = 1);
- Loop wire fracture and pontic discoloration (n = 2);
- Marginal crestal bone loss (0.19 mm; n = 1);
- Infraocclusion relative to adjacent teeth during growth (n = 2);
- Gingival inflammation due to plaque accumulation (n = 1);
- Mild diastema formation adjacent to the pontic (n = 1);
- Fracture of composite material in the temporary crown (n = 1).

Importantly, no implant failures, no cases of infection, and no interference with overall craniofacial or alveolar development were documented during follow-up. These findings support the safety and predictability of mini-implants in selected growing patients when applied within a carefully controlled clinical protocol.

Among the 17 included studies, 10 were case reports, 6 were small case series, and only 1 was a prospective clinical study. No cohort studies or randomized trials were identified.

## 4. Discussion

This narrative review confirms the increasing clinical interest in using orthodontic mini-implants as temporary prosthetic anchors for managing tooth agenesis in skeletally immature patients. Although the data are largely descriptive, the reports consistently show favorable outcomes in terms of implant stability, patient satisfaction, and alveolar bone preservation during growth.

Compared to conventional approaches—such as adhesive bridges, removable retainers, or orthodontic space closure—mini-implants offer multiple clinical advantages. Their small diameter and lack of osseointegration allow for immediate functional loading, atraumatic removal after growth completion, and re-insertion of definitive implants without compromising the alveolar crest. Moreover, they facilitate optimal esthetic positioning of temporary crowns, which is particularly critical in the anterior maxillary region [8,9,13].

Wilmes et al. reported a 95.2% clinical success rate in their series of 21 mini-implants used to support temporary crowns, with the only failure occurring in the posterior region near a molar. Based on this, the authors recommended avoiding placement in posterior segments and emphasized the importance of selecting larger implant dimensions (2 × 11–15 mm) and preventing premature occlusal contacts to minimize functional overload [13]. A key benefit reported across studies is the maintenance—or even gain—of alveolar bone volume, contrasting with the bone resorption typically observed in prosthetic gaps left unfilled or restored with passive appliances. Wilmes et al. described radiographic stability of the ridge over long-term follow-up [13], Jardim et al. documented 2.2 mm of vertical bone gain [19], and Lee et al. observed only 0.19 mm of marginal bone loss over a 24-month period [8]. These findings suggest that mini-implants may not only serve esthetic purposes but also preserve the biological environment necessary for future prosthetic rehabilitation. Similarly, Sarkar et al. and Ragulakollu et al. emphasized the dual benefit of tissue support and space preservation using this technique [11,20].

Although current clinical applications of mini-implants are largely based on titanium surfaces, future advances may include the use of bioactive coatings and biologically enhanced interfaces to further optimize tissue response and implant performance. Recent studies have shown promising results for surface modifications using hydroxyapatite, calcium phosphates, silver, or nanostructured layers, as well as the incorporation of biological agents such as growth factors, osteogenic peptides, or antimicrobial molecules. These strategies aim to promote osseointegration, reduce microbial colonization, and improve the healing process, which are particularly relevant for a pediatric population with high tissue plasticity and prolonged treatment timelines [21,22].

Nevertheless, complications have been reported. Jofré and Werner noted repeated dislodgement of the temporary crown and difficult explantation due to partial osseointegration (i.e., permanent bone integration of the implant, which may complicate later removal), which caused damage to the alveolar ridge [7]. Gianetti et al. and Sfeir et al. also observed osseointegration, raising concerns about long-term biocompatibility and the possible shift toward bone integration despite smooth surface design [4,16]. Ragulakollu et al. reported fracture of the composite pontic [20], and Negi et al. noted gingival inflammation related to hygiene [1]. These complications highlight the importance of careful prosthetic design and follow-up.

The issue of vertical adaptation during growth has also been explored. Cope and McFadden described controlled unscrewing of the implant to allow compensation for skeletal development, achieving 8 years of successful function without infraocclusion (i.e., the vertical misalignment of the implant crown due to continued eruption of adjacent teeth during growth) [14]. In contrast, Abozena et al. reported infraposition of the pontic in two patients [17]. However, most authors, including Lee et al., Rathi et al., and Jardim et al., did not report significant infraocclusion [8,9,19]. One explanation may be the limited vertical growth potential of the alveolar process in congenital agenesis sites, which could reduce the risk of vertical discrepancy. Alternatively, it may reflect relatively short observation periods or the use of passive pontic designs.

A particularly creative biomechanical solution was presented by Ciarlantini and Melsen [15], who used a transverse mini-implant anchoring a loop-activated pontic, which was reactivated every four weeks. This method aimed to stimulate soft and hard tissue adaptation in the anterior maxilla, thereby reducing the risk of infraposition.

The included studies used a wide range of imaging protocols. Most studies relied on panoramic and periapical radiographs, while CBCT was performed in 12 out of 42 patients (28.6%), including studies by Sfeir et al., Jardim et al., Sarkar et al., and Rathi et al. [4,11,14,19]. A lack of standardized imaging—particularly 3D volumetric follow-up—limits direct comparison of outcomes and prevents robust quantitative evaluation of bone remodeling around implants. This heterogeneity in imaging modalities limits the comparability of outcomes and may lead to under- or overestimation of bone preservation. Future studies should adopt standardized radiographic protocols, ideally including serial CBCT or calibrated periapical radiographs, to allow for reliable volumetric or dimensional assessment of alveolar bone changes over time.

Despite these positive clinical findings, the limitations of the existing evidence must be acknowledged. The available studies consist predominantly of case reports and small case series, lacking control groups, blinding, or prospective protocols. Follow-up duration varied, and many outcomes were subjectively assessed without validated esthetics or functional scoring systems. Additionally, radiological protocols were inconsistent, and CBCT was underutilized. These methodological shortcomings preclude meta-analytic synthesis and weaken the strength of clinical recommendations.

Nonetheless, the collected evidence suggests that mini-implants constitute a safe, minimally invasive, and reversible therapeutic option for interim prosthetic rehabilitation in growing individuals. They enable space and tissue maintenance, restore esthetics and function during critical phases of development, and preserve conditions favorable for definitive implant placement. Future studies should focus on prospective multicenter designs, adopt standardized imaging and outcome assessment protocols, and clarify long-term biological behavior, particularly regarding the risk of osseointegration and infraocclusion. The overall level of evidence across the included studies is low. Most publications were case reports or small case series, often without control groups, blinding, or standardized outcome assessment. Only one prospective study was identified, and no cohort or randomized trials were available. This reflects the exploratory nature of the field and highlights the urgent need for higher-quality clinical investigations. Until such data are available, conclusions regarding clinical protocols should be interpreted with caution.

Due to the heterogeneity and descriptive nature of the available evidence, this narrative review does not allow for clinical guidelines or treatment recommendations. Its primary goal is to map the current scope of published clinical experiences and identify areas requiring structured research. Nonetheless, the collected evidence suggests that mini-implants constitute a safe, minimally invasive, and reversible therapeutic option for interim prosthetic rehabilitation in growing individuals. They enable space and tissue maintenance, restore esthetics and function during critical phases of development, and preserve conditions favorable for definitive implant placement.

While this review offers an overview of the currently available reports on orthodontic mini-implants for interim tooth replacement in growing patients, the limited number of cases, the heterogeneity of treatment protocols, and the predominance of descriptive data preclude the formulation of clinical recommendations. Instead, the findings underscore the urgent need for prospective, multicenter studies with standardized methodology. Future research should clarify indications, optimize clinical protocols, and develop validated outcomes to support evidence-based decision-making.

## 5. Conclusions

Orthodontic mini-implants may represent a safe, effective, and reversible option for interim prosthetic rehabilitation in skeletally immature patients with hypodontia or traumatic tooth loss. Available clinical reports suggest that their use may support space maintenance, esthetic restoration, and preservation of alveolar structures during craniofacial growth. However, further high-quality studies are warranted to confirm their long-term safety, functional outcomes, and optimal clinical protocols.

## Figures and Tables

**Table 1:** Summary of clinical studies on the use of orthodontic mini-implants for interim prosthetic rehabilitation in growing patients with hypodontia or traumatic tooth loss. The table presents patient demographics, etiology of tooth absence, implant sites, imaging modalities, follow-up duration, clinical outcomes, and reported complications.

Author(s), Year, Country	Study Design	Number of Cases (Age, Sex)	Type of Tooth Deficiency	Planned Treatment and Mini-Implant Type	Implant Placement	Follow-Up Duration	Outcome	Imaging Modality	Complications
1. Wilmes B et al., 2014, Germany [13]	Case series	3; 13–14 years, 2 female, 1 male	Hypodontia 12, 22	Crowns on titanium mini-implants 2 × 13. 2 × 11 mm, Benefit	12, 22	4–5 years	No clinically significant bone resorption	Panoramic radiograph	None
2. Cope JB, McFadden D, 2014, USA [14]	Case series	2; 12, 13 years; 1 male, 1 female	Hypodontia of tooth 12	Polycarbonate crown on titanium mini-implant 2.2 mm, MSI IMTEC Sendax MDI MAX, 3M Unitek, placed with full-thickness flap	12	Case 1: 99 months (≈ 8 years); Case 2: 27 months	Positive—no infraocclusion, implant stable, ~1 mm vertical bone gain	Panoramic and periapical radiographs	None
3. Sfeir E. et al., 2014, Lebanon [4]	Case series	3 patients; 11–12 years; 3 males	Oligodontia associated with ectodermal dysplasia	Composite crowns followed by ceramic crowns on titanium mini-implants, 1.8–2.4 × 13 mm MDI, 3M ESPE, placed with full-thickness flap	Maxilla + mandible (anterior part)	1–4 years	Positive—no clinically relevant bone resorption, good prosthetic stability, no infraocclusion, evidence of osseointegration	CBCT, periapical, panoramic and bite-wing radiographs	None
4. Jofré J., Werner A., 2015, Chile [7]	Case report	1; 10 years; male	Avulsion of tooth 21	Acrylic crown on titanium mini-implant, 1.8 × 14 mm, MTI Transitional Implants, Dentatus, NY, USA	21	6 years	Negative—vertical alveolar growth prevented crown retention after 6 years; mini-implant had to be removed	Cephalometric and panoramic radiographs	Crown dislodged 6 times; removal required >50 N cm force and caused significant alveolar bone damage
5. Kilic S. et al., 2015, Turkey [5]	Case report	1; 6 years; male	Mandibular anodontia in ectodermal dysplasia	Ball-retained overdenture on two titanium mini-implants, 2.9 × 13 mm, Endure MDI/ImtecCorpR	Mandible, anterior part	6 years	Positive—no notable bone resorption, normal mandibular growth	Cephalometric and panoramic radiographs	None
6. Kalia A.J., 2015, India [3]	Case report	1; 16 years; female	Hypodontia of tooth 12	PFM crown on titanium mini-implant, 1.4 × 10 mm, AbsoAnchor	12	12 months	Positive—no significant bone resorption	Panoramic and periapical radiographs	None
7. Ciarlantini R., Melsen B., 2017, Italy [15]	Case series	5; 10–13 years; sex not specified	Hypodontia of teeth 12, 22	Composite pontic bonded to a stainless-steel loop fixed to a titanium mini-implant, size not specified, Aarhus, Medicon Instrumente, Tuttlingen, Germany	12, 22	5 years	Positive—alveolar ridge growth maintained (palatal screw)	Periapical radiographs; CBCT in 2 patients	Loop fracture in 1 patient; pontic discoloration in 1 patient
8. Oliveira N. et al., 2017, Brazil [6]	Case report	1; 10 years; male	Avulsion of teeth 11 and 21	Acrylic crowns on titanium mini-implants, 1.6 × 10 mm, Morelli, Sorocaba, São Paulo, Brazil	11, 21	12 months	Positive—no appreciable bone resorption	Panoramic radiograph every 3 months	None
9. Giannetti L. et al., 2020, Italy [16]	Case report	1; 8 years; male	Avulsion of 11 at age 8 and 21 at age 10	Initial composite crowns, replaced after 1–3 yrs by ceramic crowns on titanium mini-implants, 2.5 × 13 mm, manufacturer not specified	11, 21	Clinical/radiographic controls at patient ages 13 and 15 yrs	Positive—no measurable bone resorption; clear evidence of osseointegration	CT scan and panoramic radiographs	None
10. Sarkar N. et al., 2021, India [11]	Case series	2; 12 years; 1 male, 1 female	Hypodontia of teeth 21, 22	Acrylic crowns on mini-implants 1.5 mm × 11 mm, material and manufacturer not specified	21, 22	5–15 months	Positive—no clinically significant bone resorption	Panoramic radiograph	None
11. Lee B.J. et al., 2021, South Korea [8]	Case report	1; 14 years; male	Hypodontia of teeth 25, 34, 35, 44	Unrestored titanium mini-implant 1.6 mm × 7 mm, ORLUS ET; Ortholution Co., Seoul, Korea)	44	2 years	Slight vertical bone loss (0.19 mm at 24 months)	Panoramic and periapical radiographs	0.19 mm marginal bone loss over 24 months; no other adverse events
12. Abozena N. et al., 2022, Egypt [17]	Prospective study	15; 11–13 years; sex not specified	Avulsion / congenitally missing incisors	PFM crowns on titanium IMPLA mini-implants, 3 mm × 11.5 mm, 3 mm × 13 mm, Schütz Dental GmbH)	12, 11, 21, 22	24 months	Positive—no clinically significant bone resorption	Periapical radiographs every 6 months	Two cases of 2 mm infraocclusion; no apparent inhibition of maxillary alveolar bone growth
13. Negi S. et al., 2022, India [1]	Case series	2; 13-year-old female, 14-year-old male	Avulsion 11, 21	PFM crowns on titanium mini-implants,1.5 mm × 12 mm, SK Surgicals, Pune, Maharashtra	11, 21	12 months	Positive—no clinically significant bone resorption	Periapical radiographs	Gingival inflammation around one mini-implant at 3 months (poor oral hygiene)
14. Saha N. et al., 2023, India [18]	Case report	1; 12 years; female	Hypodontia 12, 22	Composite crowns on titanium mini-implants 1.5 mm × 10 mm, SK Surgicals	12, 22	9 months	Positive—no clinically significant resorption	Panoramic and periapical radiographs	Mild 1 mm diastema
15. Jardim A.F.V. et al., 2023, Brazil [19]	Case report	1; 14 years; male	Hypodontia 12	Acrylic crown on titanium mini-implant, 2 mm × 10 mm, Morelli, Sorocaba, Brazil	12	3 years	Positive—2.2 mm vertical bone gain, 0.3 mm reduction in buccolingual width	CBCT follow-up	None
16. Ragulakollu R. et al., 2023, India [20]	Case report	1; 13 years; male	Hypodontia 45	Composite crown on stainless-steel mini-implant, 2 mm × 14 mm, SK Surgicals IZC, placed with full-thickness flap	45	12 months	Positive—no clinically significant bone resorption	CBCT, panoramic radiograph	Minor chipping of composite on buccal crown surface, polished smooth
17. Rathi N.V. et al., 2023, India [9]	Case report	1; 12 years; female	Hypodontia 22	Composite crown on titanium mini-implant, 2.5 mm × 10 mm, Osstem MS Transitional Implant, placed with full-thickness flap	22	24 months	Positive—no clinically significant bone resorption	CBCT, periapical radiographs	None

## Data Availability

The data presented in this study are available on request from the corresponding author. The data are not publicly available due to privacy restrictions.
